# Modeling the Human Bone–Tumor Niche: Reducing and Replacing the Need for Animal Data

**DOI:** 10.1002/jbm4.10356

**Published:** 2020-03-23

**Authors:** Srinivasa R Rao, Claire M Edwards, James R Edwards

**Affiliations:** ^1^ Botnar Research Centre, Nuffield Department of Orthopaedics, Rheumatology and Musculoskeletal Sciences University of Oxford Oxford UK; ^2^ Nuffield Department of Surgical Sciences University of Oxford Oxford UK

**Keywords:** ANIMAL MODELS, BONE MODELING, BONE REMODELING, CANCER, TUMOR‐INDUCED BONE DISEASE

## Abstract

Bone is the most common site for cancer metastasis. Understanding the interactions within the complex, heterogeneous bone–tumor microenvironment is essential for the development of new therapeutics. Various animal models of tumor‐induced bone disease are routinely used to provide valuable information on the relationship between cancer cells and the skeleton. However, new model systems exist that offer an alternative approach to the use of animals and might more accurately reveal the cellular interactions occurring within the human bone–tumor niche. This review highlights replacement models that mimic the bone microenvironment and where cancer metastases and tumor growth might be assessed alongside bone turnover. Such culture models include the use of calcified regions of animal tissue and scaffolds made from bone mineral hydroxyapatite, synthetic polymers that can be manipulated during manufacture to create structures resembling trabecular bone surfaces, gel composites that can be modified for stiffness and porosity to resemble conditions in the tumor–bone microenvironment. Possibly the most accurate model system involves the use of fresh human bone samples, which can be cultured ex vivo in the presence of human tumor cells and demonstrate similar cancer cell–bone cell interactions as described in vivo. In addition, the use of mathematical modeling and computational biology approaches provide an alternative to preliminary animal testing. The use of such models offers the capacity to mimic significant elements of the human bone–tumor environment, and complement, refine, or replace the use of preclinical models. © 2020 The Authors. *JBMR Plus* published by Wiley Periodicals, Inc. on behalf of American Society for Bone and Mineral Research.

## Introduction

Bone is the most common site for tumor metastasis, particularly in prostate cancers where approximately 70% of patients dying of these cancers show evidence of metastatic bone disease postmortem. Tumor‐induced skeletal disease may also complicate a wide range of other malignancies (thyroid, renal, and lung cancers; melanoma), hematological cancers (multiple myeloma), or primary bone tumors, which develop within the skeleton, resulting in considerable morbidity and complex demands on health care resources worldwide.^1^

There are a large number of different animal models used for the study of tumor‐induced bone disease. These include models where human or mouse tumor cells are placed at extraosseous sites, including the primary sites of tumor (eg, breast),^2^ where tumor cells are inoculated directly into the circulatory system (eg, heart, tail vein),^3^, ^4^ or where tumor cells are implanted directly into skeletal sites (eg, through the tibial plateau).^5^

Our previous work and that of others, has shown that tumor cells interact directly with cells of the bone environment in models of cancer–bone disease, which can be visualized and quantified histologically to assess the effects of drug treatment upon the bone–tumor niche.^4^, ^6^ Furthermore, the volume of bone destruction can be accurately determined by high‐resolution μCT scanning, overall tumor volume assessed by soluble tumor‐derived factors released into the serum, and through the visualization of fluorescently tagged cancer cells.

In vitro studies employing cancer cells in 2D culture do not mimic the in vivo environment or account for the contribution of the many cellular components that comprise the tumor–bone niche and contribute to the “vicious cycle” of cancer–bone disease, particularly osteoclasts but more recently osteoblasts, adipocytes, immune cells, and stromal components are also thought to act. Similarly, limitations and confounding factors introduced by the use of murine in vivo bone–tumor models include the species‐specific differences between human and mouse, such as a significantly faster rate of bone turnover in the latter, and the use of young animals with developing skeletal components versus the mature human skeleton as the typical site of secondary tumor development. Also, a mixed species approach is common in preclinical models of cancer–bone disease, where human cells are inoculated into immunocompromised mice to avoid rejection of the cancer cells by the host. The important contribution of the immune system within the bone–tumor niche is lost in such models, a feature that our ongoing work has highlighted.^7^

Despite limitations in their effectiveness and interpretation of results, rodent models of bone remodeling, including cancer–bone disease, continue to represent the most popular approach to the study of the complex multicellular bone microenvironment in situ. There are, however, a large number of alternate in vitro models that aim to capture the principal interactions of this system, and that are beginning to form a valuable and accurate assessment tool capable of reducing the use of animals, in some instances, replacing animal models entirely. Furthermore, the high throughput and lower costs of such approaches offer an attractive and cost‐effective means to accurately and reliably gather preliminary information on new drug compounds or explore molecular mechanisms of action, to reduce, replace, and refine further preclinical studies.

### Culture model systems

In its simplest form, the use of 2D coculture systems has shown how tumor cells and the release of cancer‐derived products impact bone cells when cultured collectively. Similarly, the nature of the cancer–bone microenvironment, where invading tumor cells hijack the delicate relationship between bone‐forming osteoblasts and bone‐resorbing osteoclasts (and other contributing cell types of the bone niche), indicates how bone cells also impact the growth, survival, and activity of tumor cells.^8^ This “vicious cycle” of tumor‐induced bone disease is fueled by the communication between tumor cells and the cells of the bone marrow niche and has been demonstrated in simple 2D cultures using a variety of tumor cell types, including breast, prostate, and myeloma. However, certain physiological conditions, including an absence of bone matrix and associated proteins as well as a structural architecture resembling bone, contribute to the poorly modeled environment that must be considered in the interpretation of such information.

The use of 3D culture models, including a surrogate “bonelike” structure such as type I collagen, hydroxyapatite scaffolds, or biodegradable polymers, offer a more realistic environment within which bone and tumor cells are exposed to and stimulated by the physical parameters of a 3D structure. The heterogeneous microenvironment of a human tumor in situ is also more accurately recreated in 3D, where cells at the leading edge of a tumor are more metabolically active, benefiting from increased availability of nutrients and oxygen in contrast to cells at the tumor core, which are hypoxic and nutrient‐starved. Since their introduction in the early 1970s by Sutherland and colleagues,^9^ the model we now recognize as spheroid cultures have come to represent a popular in vitro model of multicellular tumor microenvironments, including that of bone. This is based, in large part, to their capacity to mimic a heterogeneous cellular niche, incorporate matrix components, and demonstrate a similar response to stimuli within the surrounding media as seen in the in vivo tumor, including pro‐ or antitumor therapies and cytokines.^10^

The material basis for spheroid culture has relied heavily upon the use of naturally occurring matrices, most notably collagen‐based gels (eg, using rodent type 1 collagen) or the gelatinous protein mixture known commercially as Matrigel (derived from Engelbreth–Holm–Swarm mouse sarcoma cells).^11^, ^12^, ^13^, ^14^

The use of natural matrix‐derived products encourages a strong cellular adherence to the surrounding matrix, coupled with an uninhibited growth pattern, leading to the popular use of such materials as surrogates to soft tissue in situ and also the bone–tumor microenvironment. Typical procedures include the initial culture and expansion of required cells in 2D, followed by encapsulation in a gel matrix (eg, alginate, collagen) brought about following polymerization of cell–matrix droplets in a calcium chloride solution. Cell spheroids can then be cultured in an appropriate media for days to weeks, after which 3D‐cell growth and matrix interactions can be assessed by processing for histology and tinctorial or immunohistochemical staining, or isolated following incubation in an EDTA buffer solution and centrifugation for further molecular analysis.^15^, ^16^, ^17^ Organic matrices are, however, limited by the heterogeneity that exists between the variable sample preparations, and where a more functionally and architecturally defined matrix offers additional elements of the host microenvironment not well‐modeled in natural hydrogels, such as structural continuity and defined variations in matrix stiffness.

The use of synthetic matrices offers the potential to overcome such obstacles through the creation of cellular environments tailored to the specific in vivo characteristics being modeled. Ashworth and colleagues have described a self‐assembling peptide gel that can be customized with further matrix components if desired, and levels of stiffness modified through a strictly controlled gelation process.^18^ The initial formation of a matrix‐free precursor is achieved using a commercially available peptide gelator containing phenylalanine, glutamic acid, and lysine, the concentrations of which dictate the overall stiffness of the matrix product. Homogeneity within the gel matrix is ensured by first reverting to a liquid state brought about by the manipulation of pH and temperature (heated to 80°C); after which, cells and other organic components can be added.

Similar hybrid 3D‐spheroid cultures have been developed using fragmented fibers from electro‐spun materials that mimic the extracellular matrix. Enzyme‐digested sheets of poly‐L‐lactide (PLLA) are coated with polydopamine, or alternatively biomineralized with a sodium hydrogen carbonate solution, to more fully model the architecture and stiffness of the bone environment.^19^, ^20^ Such model systems show improved viability and function of stem cells and enhanced differentiation toward an osteogenic lineage. Moreover, mineralized spheroid cultures can fuse to form a bonelike 3D‐tissue construct, which maintains cell growth and distribution of mineral throughout the structure.^20^

Hard‐scaffold materials have also been used to mimic the bone or extracellular matrix environment. These include the use of synthetic apatite ceramic and natural calcite, along with such diverse biological substances as elephant tusk dentine, hen egg, and oyster and slipper shells.^21^, ^22^ The use of silk as a scaffold material has been used to model the tumor environment of principal bone‐metastasizing tumors such as prostate^23^ and breast^24^ cancers, as well as multiple myeloma.^25^ Trabecular bonelike structures can be created from silk fibroin extracted from silkworm (*Bombyx mori*) cocoons following a boiling, purifying, and drying procedure, which can then be seeded and successfully populated with bone‐forming osteoblasts along with selected tumor cells.^26^

Synthetic polymers in combination with bone mineral constituents have also proved successful as trabecular bone mimetics to model the metastasizing cancer environment. Use of a reactive polyurethane material combined directly with the mineral component of bone known as hydroxyapatite, has been used to form a hybrid composite consisting of lysine methyl ester diisocyanate, nanocrystalline hydroxyapatite, and polycaprolactone triol catalyzed by an iron acetylacetonate solution. The bonelike construct is formed through the 3D printing of the polyurethane–hydroxyapatite material using defined patterns based upon μCT scanning of human trabecular bone. The resulting scaffold demonstrates a mineral content comparable to that of human bone, within which human bone marrow‐derived stem cells attach, proliferate, and differentiate into active mineralizing osteoblasts and survive in coculture with seeded cancer cells.^27^, ^28^

Recently, novel approaches to material design have created diverse structures upon and within which a cellular niche, including a cancer–bone cell niche, might be created. The development of 3D‐microfiber scaffolds using melt electrowriting technology (MEW) is one approach capable of mimicking both the structural and chemical environment.^29^ Molten polycaprolactone is used to first generate a layered 3D structure. This melt electrowriting technique sprays polycaprolactone over a period of 2 days, after which a laser‐cutting procedure refines bonelike scaffold structures with fiber diameters approximately 12 μm in diameter, spaced approximately 150 μm apart. Bonelike properties are conferred by coating with calcium phosphate prior to cell seeding.

A variety of bioreactor approaches have also aimed to recapitulate the bone–tumor niche in 3D. These include the seeding of tumor cells or bone stromal populations with type I collagen‐coated dextran beads, which are collectively cultured in a microgravity‐simulated culture achieved using a rotating‐wall vessel.^30^ The resulting tumor organoid proliferates in the rotary culture system as an in vivo cancer metastasis, is responsive to extracellular stimuli, and can be transplanted in vivo if necessary.

Tumor–bone cell 3D cultures can also be achieved in the absence of the rotating vessel, but where a continual external cellular pressure exerted by fluid flow is retained. The dialysis bioreactor approach proposed by Krishnan and colleagues supports the growth and coculture of different cell types, the efficient exchange of cytokines, growth factors, and removal of toxic waste products, combined with the production of a 3D‐extracellular matrix.^31^ This approach consists of a distinct cellular compartment and reservoir of growth medium separated by a 6‐ to 8‐kDa dialysis membrane and where cell populations can be cultured directly at the bottom of the gas‐permeable film. Metabolic waste products of low molecular weight (eg, lactic acid) continuously dialyze out of the growth space into the media reservoir against a concentration gradient, whereas low‐molecular‐weight nutrients (eg, hexose, amino acids) dialyze into the growth space from the reservoir. Following the initial culture periods (up to 60 days), bone‐forming osteoblasts produce a 3D‐collagenous‐extracellular matrix >20‐μm thick, which can be modified and quantified as a measure of osteolysis‐like activity by the presence of osteoclasts and/or tumor cells. Coculture of 60‐day osteoblast‐derived matrix with bone‐resorbing osteoclasts reduced matrix thickness by 31%. Interestingly, triculture with bone metastatic MDA‐MB‐231 breast cancer cells further reduced matrix thickness by over 40%.

**Figure 1:**
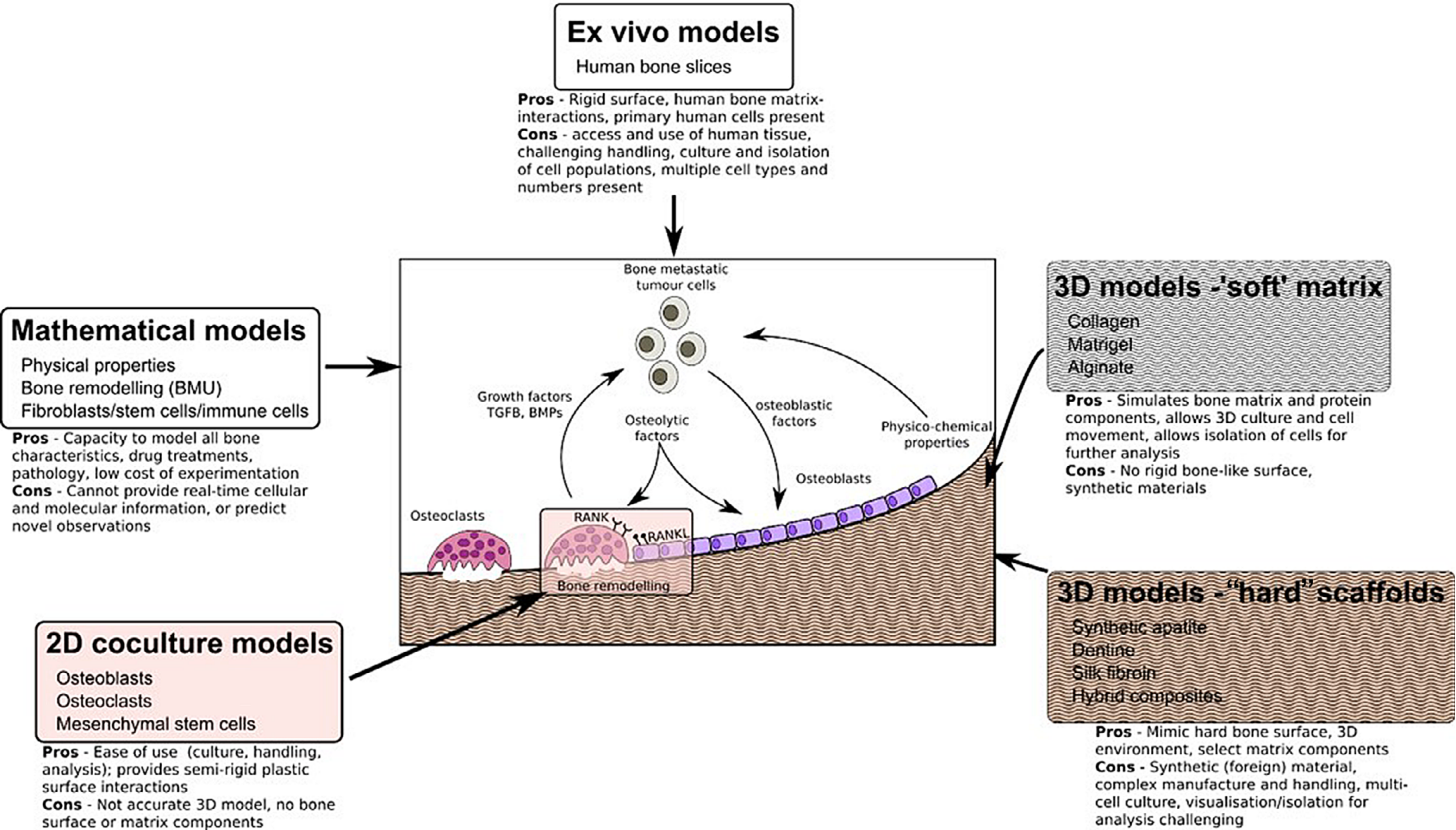
Summary models for animal replacement in modeling the tumor–bone niche. The computational modeling and inclusion of tumor cells in synthetic and organic hard and soft matrices, including human bone and local host cells, offers alternate approaches to the use of animals in the study of cancer–bone disease.

The capacity to represent the large number of cell types present within the bone–tumor microenvironment within an in vitro model system represents a significant challenge to the recapitulation of this unique and complex niche in culture systems. Varying numbers of over a dozen different cell types could be included if the true cellular skeletal environment was to be fully recreated, including endothelial cells, fibroblasts, adipocytes, osteocytes, and an array of immune cells. The in vitro culture of all cell types at the same time and in the same place, would likely result in a particularly disorganized and chaotic system, the data from which would be difficult to interpret. This does, however, highlight the remarkable control that the skeletal environment manages over this complex cellular and matrix network. In attempting to reproduce the specific cross‐talk among several cell types, including cancer cells, while retaining a matrix component, microfluidic devices have been employed. These offer the opportunity to culture different cell types in temporary and separate environments, which might also be connected or layered together over the growth period. Such a model was employed to integrate a vascular component to 3D‐bone–tumor culture, an essential component of the metastatic and tumor proliferative process, but which is particularly difficult to model in vitro. Bersini and colleagues propose a triculture microfluidic 3D system that also includes a type I collagen gel matrix.^32^ Mesenchymal stem cells derived from human bone marrow are predifferentiated in osteogenic medium over 2 weeks to stimulate commitment toward an osteoblast lineage. These cell populations embedded in a collagen matrix are introduced into a microfluidic channel; endothelial cell lines are seeded 3 days later within a distinct central media channel to allow for the creation of a monolayer of endothelium between the channel walls and the gel‐channel interfaces over a 6‐day period. Following this, tumor cells can be added and extravasation and micrometastases between vascular and bone channels assessed, along with changes in cellular expression induced by the combined culture environment and the contact with different cell types (Fig. 1).

### Ex vivo bone models

The diverse and creative development of culture systems to model in situ human bone environments has led to the use and application of a variety of assays. However, the use of primary bone tissue in such experiments offers a direct way of sampling the skeletal milieu and where the introduction of new or metastasizing cell types can be easily achieved. ex vivo assays using fresh bone such as fetal rat long bones,^33^ mouse calvariae,^34^, ^35^ or embryonic metatarsals,^36^ have previously been used successfully to investigate cell‐specific and heterogeneous populations to test potential bone anabolic agents or to study vascular differentiation and growth.

Previous attempts to model the tumor–bone niche using fresh bone tissue have employed murine calvariae dissected from postnatal mice (up to 7 days). Bisected calvariae preincubated for 24 hours in media were subsequently cultured on metal grids in close proximity to, but not touching, cancer cells that were cultured in monolayer below. This two‐compartment system allows for paracrine signaling between the fresh bone and tumor cells and where changes in resorption within the bone (assessed by collagen breakdown and release) could be quantitated, along with alterations in molecular and protein expression.^37^ This model has been developed further to culture tumor cells in direct contact with the calvarial bone surface.^38^ Similarly, fresh bisected postnatal calvariae can be cultured in the presence of tumor cells in a rotating culture system where direct contact is stimulated and bone–tumor cell interactions can be recorded. Media constituents can be altered to model bone‐resorbing or bone‐forming systems.^39^

**Figure 2:**
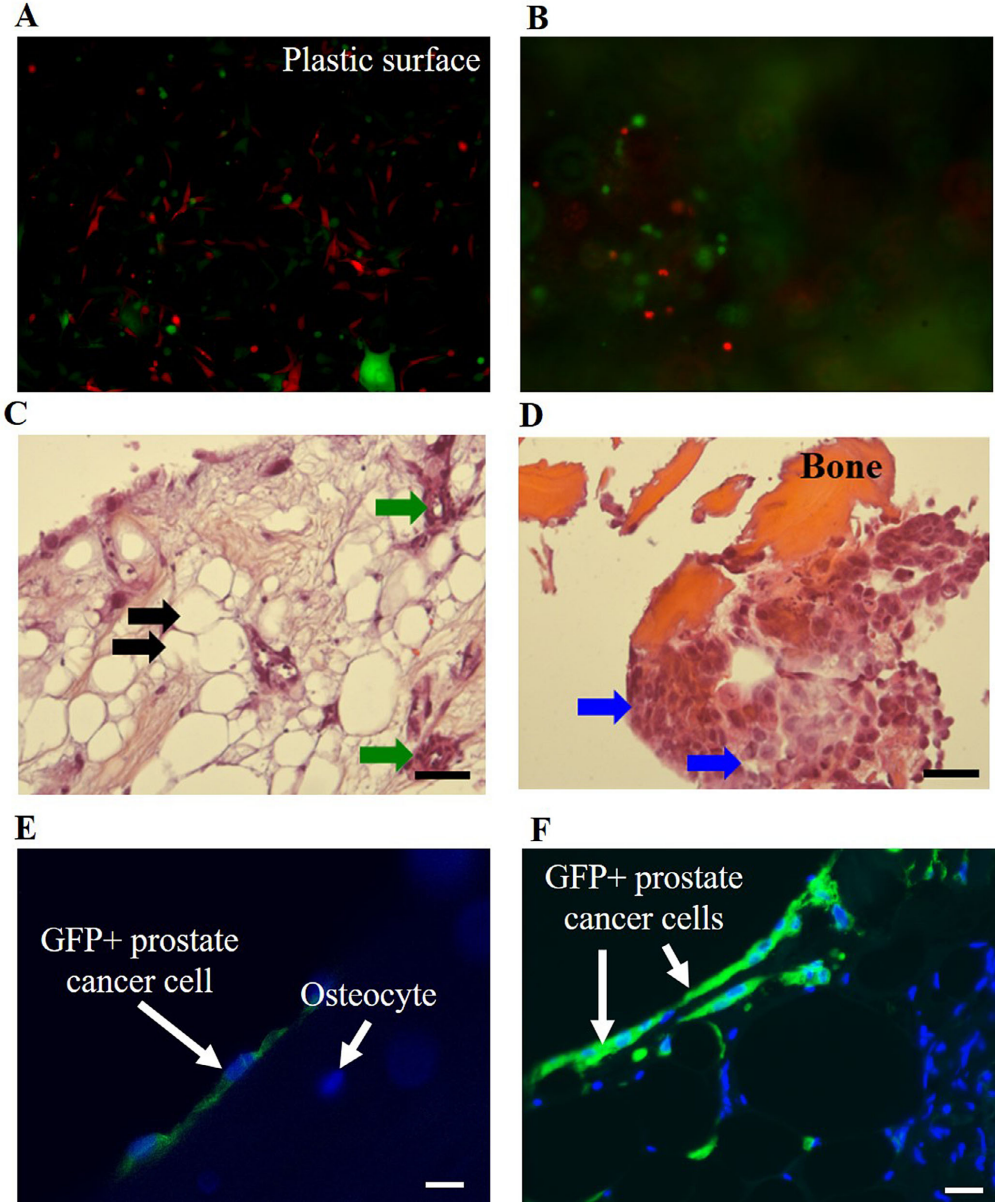
Ex vivo human bone–tumor cell niche. Viable enhanced green fluorescent protein‐ (EGFP‐) tagged PC3 tumor cells (green) and mcherry‐labeled HS5 stromal cells (red) are visualized equally live on 2D plastic surface (*A*, scale bar = 200 μm) and live in situ within the human bone core assay (*B*, scale bar = 200 μm). Viable normal bone matrix and marrow networks are visible following culture up to day 14 (*C*, scale bar = 500 μm), allowing for the continued growth of niche populations including adipocytes (black arrows) and vascular components (*C*, green arrows) and the propagation of inoculated tumor cells adhering and proliferation upon and within bone (blue arrows) (*D*, scale bar = 200 μm) (H&E‐orange G), and confirmation of EGFP tumor cells interacting with bone cells of the host tissue by immunofluorescent staining (*E*, *F*, scale bar = 50 μm).

Holen et al. have recently extended this model system to more accurately focus this system upon the human bone–tumor niche where fresh human trabecular bone samples are used in place of dissected intramembranous bone from rodents. Cores of human bone isolated from the fresh femoral head of consented patients following replacement surgery for arthritic disease, were obtained and uniform discs of fresh bone samples prepared (0.5‐cm diameter × 0.5‐cm thick) from uninvolved, normal bone regions. Representative control discs from each core are assessed for average bone volume and trabecular parameters, with remaining discs of human bone cultured under experimental conditions. This includes the coinoculation with human cancer cell lines and culture over 7 to 14 days. Bone discs from the human ex vivo assay can be analyzed histomorphometrically to determine cellular distribution within and around human trabecular bone structures of both the existing host cells of the human bone sample and the introduced human tumor cells, along with changes in bone volume, and tumor‐ and bone‐derived release of soluble factors. Prostate cancer cells inoculated into fresh human bone were shown to adhere to trabecular bone regions during the culture period, forming independent tumor colonies similar to that observed in human metastatic disease, and appeared viable upon immunofluorescence staining, similar to those cultured in 2D conditions on tissue culture plastic (Fig. 2A–B). Prostate cancer cells were clearly visible among bone marrow stromal cells on tissue culture plastic and also following implantation within bone, interacting directly with the bone matrix and in contact with primary bone cells lining the bone surface (Fig. 2C–D), and invading the cellular microenvironment and trabecular bone structure of the bone core slices (Fig. 2E–F).

Similarly, a humanized bone–cancer model was described by Holen and colleagues,^40^ where mouse and human breast cancer cells were seeded upon discs of viable human subchondral bone. In addition to assessments of tumor cell growth and changes in bone volume as described above, humanized bone discs can also be successfully transplanted in vivo if necessary.^40^

### Mathematical and computational modeling

A further expansion of how the interactions between cancer cells and the cellular and matrix components of the skeleton might be modeled is through the use of computational modeling. Although all the models described so far allow for the study of simple cell–cell interactions in the presence/absence of different local constituents (eg, growth media, resorptive factors, drugs), more complex mechanisms, such as interactions between increased numbers of multiple cell types, reaction kinetics, and diverse microenvironment changes, are difficult to model using cell culture. Mathematical and computational models of biological systems might be employed to bridge this gap.

Existing published data describing the basic parameters of a single cell (size, force exerted by cell processes on the substrate, rates of secretion of enzymes and growth factors) and the interaction of different signaling molecules can be collated with varying levels of abstraction. Such a systems biology approach might be adopted in modeling the single tumor cell within a bone environment in a manner similar to that reported for the simulation of the complete Mycoplasma genitalium cell by Karr and colleagues,^41^ where the effects of 525 genes were curated and grouped into functional modules. Previously, computational modeling has been applied to reveal mechanistic insight into cancer cell proliferation and invasion by modeling aspects such as cell division and death, hypoxia, adhesion of cancer cells to neighboring cells and the extracellular matrix,^42^, ^43^, ^44^ angiogenesis, and interaction of cancer cells with immune cells and other cells of the microenvironment. Modeling the multiple steps involved in the metastatic spread of cancer cells from the primary site and including the dissolution of the basement membrane, intravasation, dissemination through the bloodstream or lymphatic supply, extravasation and seeding, dormancy and clonal expansion, is a challenging prospect. But because several aspects of this process are impossible to measure or recapitulate in a reasonable timeframe in animal models, computational and mathematical models, such as those described by Franssen and colleagues, have the potential to reduce animal use, costs, and improve experimental efficiency.^45^

Similarly, attempts to assess bone mathematically have resulted in models describing alterations in physical properties such as bone strength,^46^ trabecular structure, permeability,^47^ and bone healing.^48^, ^49^ This approach might also be applied to the context of cancer‐induced bone disease, as demonstrated by Eggermont and colleagues who incorporated patient‐specific finite‐element analysis to predict fracture risk in individuals with bone metastasis.^50^ The bone‐remodeling process, involving bone formation and destruction by osteoblasts and osteoclasts, respectively, has been abstracted in mathematical models. The basic multicellular unit, which consists of models of these cells, has been studied in a normal context as well as in disease.^51^, ^52^, ^53^ These studies aim to interrogate the complex interplay of factors involved in the cross‐talk between the various cells of the bone microenvironment.

Well‐established key events that occur during the tumor metastasis/bone remodeling process have been used in attempts to combine the computational modeling of both the bone and tumor environments to accurately predict interactions within the cancer–bone niche. This phenomenon has been modeled in multiple myeloma‐induced bone disease, by focusing on the inhibition of osteoblasts (through promotion of apoptosis and inhibition of differentiation) and activation of osteoclasts (through modulation of RANKL:osteoprotegerin balance).^54^ Similarly, mathematical modeling has shown that myeloma cells in the bone microenvironment set‐off unstable oscillations of osteoblast and osteoclast numbers, leading to increased bone destruction.^55^ The dynamics of the tumor cell‐induced vicious cycle can also be incorporated in a mathematical model of prostate cancer–bone metastasis. This includes the modeling of bone as a reservoir of latent TGF‐β that is released and activated during tumor‐induced osteolysis, which in turn impacts the osteoblast:osteoclast balance in the remodeling unit.^56^ In addition, the role of cancer‐associated fibroblasts^57^ and immune cells^58^ in tumor cell metastasis has been modeled, which is of particular interest given the diverse cellular environment of the bone metastatic niche.

Prostate cancer has been modeled by parameterizing the initial dependence of tumor cells on androgens for growth, and their escape from this dependency in castration‐resistant cells that are also resistant to anti‐androgen therapies.^59^, ^60^, ^61^ Cook and colleagues modeled the interaction between metastatic prostate cancer cells and bone cells, which they further validated using in vivo models of PCa bone metastasis.^62^ In addition, the simulation of breast cancer cells in an extracellular matrix revealed that the activity of membrane‐bound enzyme MT1‐MMP plays a more important role in tumor invasion, compared with secreted matrix‐degrading enzymes such as MMP9.^45^ Models of breast cancer consisting of three compartments: the primary tumor, the circulatory system, and bone as the site of distant metastasis, have been proposed.^63^ Such models could be useful in determining which treatment strategies would best help to reduce metastasis by targeting the tumor cells in the circulatory compartment.

## Discussion

The use of animal models for the study of tumor‐induced bone disease is well established. A wide variety of models exist employing tumor cell types from diverse origins and host environments with a range of backgrounds, including normal healthy animals to aged or immunocompromised mice.^64^, ^65^ By our estimates, over 8000 animals/year are used to model the interactions of the tumor–bone niche worldwide, despite a range of reliable surrogates that mimic the various cellular and matrix components of this system and where the human tumor–bone niche may be recapitulated ex vivo as an alternative first‐line assay to the use of animal models in the study of cancer–bone disease.

As with all modeled systems, including animal models of human disease, there are limitations to the extent that the full physiological human environment is mimicked. Such limitations include the loss of complete bone marrow components and mechanical forces that occur in active rodent models, which might be more fully recapitulated using a modified ex vivo assay‐bioreactor approach. The use of such in vitro/ex vivo models of bone–tumor interactions may, however, provide valuable information contributing to the refinement of existing procedures in animal models and a reduction in the number of animals required, for example, in preliminary dose optimization studies where higher/lower doses with no observed effect in model systems may be excluded from any necessary further in vivo studies.

Improvements in existing models would be required to more completely represent the in vivo tumor–bone niche in one system. The capacity to model the rigid scaffold of bone, which has been shown to alter tumor cell behavior,^66^ is lost in soft tissue matrix models. The use of matrix protein components or mineralized synthetic fragments improves upon the soft, undefined architecture of 3D‐gel systems and provides pockets of mechanical stimuli resembling bone. The improved modeling of this rigidity will be important in the further development of gel and polymer spheroids. Furthermore, the capacity to effectively culture multiple cell types simultaneously is challenging and minimized in most bone–tumor models. However, the heterogenous cellular distribution of the bone microenvironment is a critical component of the tumor–bone niche and plays a diverse role in tumor establishment and development. The use of multicell cultures, such as microfluidic culture systems, offers the potential for different cell populations and matrix structures to be cultured independently or collectively and offers temporal opportunities for populations to be joined and mixed during differing culture periods. This model system is currently under investigation as a more complete mimic of the bone–tumor compartment. Similarly, fused spheroid cultures of differing cell/matrix components might also improve the in vitro modeling of the bone–tumor niche.

With increasing worldwide research efforts into cancer–bone disease, the use of model culture and mathematical systems offers a realistic and reliable alternative to the increasing use of animals to reproduce this aspect of human disease.

## Disclosures

All authors declare no conflict of interest relating to this work.
